# m6A RNA methylation in sepsis-induced cardiomyopathy: direct cardiac mechanisms, emerging therapeutic targets, and translational gaps

**DOI:** 10.3389/fmed.2026.1826751

**Published:** 2026-05-04

**Authors:** Fengmei Zhang, Lijun Zhang, Yuhan Wang

**Affiliations:** Department of Cardiovascular Surgery, West China Hospital, Sichuan University/West China School of Nursing, Sichuan University, Chengdu, China

**Keywords:** apoptosis, epitranscriptomics, ferroptosis, FTO, METTL3, N6-methyladenosine, pyroptosis, sepsis-induced cardiomyopathy

## Abstract

Sepsis-induced cardiomyopathy (SICM) develops in up to 60% of patients with sepsis, substantially increases mortality, and currently lacks specific pharmacotherapy. N6-methyladenosine (m6A), the most prevalent internal modification on eukaryotic mRNA, dynamically regulates transcript fate through coordinated actions of methyltransferases (writers), demethylases (erasers), and binding proteins (readers). Direct cardiac studies have now implicated multiple m6A regulators in several key cellular processes relevant to SICM, including inflammatory injury, apoptosis, pyroptosis, ferroptosis, and adaptive mitophagy. Current evidence highlights pronounced context dependence: the eraser FTO shows the most consistent cardioprotective profile across inflammation, ferroptosis, and mitophagy, whereas the other eraser ALKBH5 paradoxically promotes pyroptotic injury, and the writer METTL3 predominantly drives damage through distinct transcript–reader axes. Notably, several independent m6A pathways converge on the SLC7A11/GPX4/NRF2 antioxidant network, suggesting that ferroptosis-centered m6A regulation may represent the most coherent translational entry point identified thus far. This review synthesizes the direct cardiac evidence using a process-oriented framework, distinguishes injury-associated pathways from adaptive mitochondrial quality control, and identifies critical translational gaps—including heavy reliance on lipopolysaccharide-based models, limited use of primary cardiomyocytes and cardiac-specific genetic tools, and the absence of human validation—that must be addressed before m6A-targeted strategies can advance toward clinical application.

## 1 Introduction

Sepsis is a life-threatening organ dysfunction caused by a dysregulated host response to infection (1). Globally, an estimated 48.9 million cases and 11 million sepsis-related deaths occur each year (2). The heart is among the organs most vulnerable to sepsis: myocardial dysfunction develops in 40–60% of septic patients and is independently associated with a two- to threefold increase in mortality (3, 4). The pathophysiology of SICM involves inflammatory cytokine-mediated cardiac depression, mitochondrial dysfunction, dysregulated calcium handling, and the activation of multiple programmed cell death pathways, including apoptosis, pyroptosis, and ferroptosis (5, 6). Despite these mechanistic advances, no SICM-specific pharmacotherapy has been established, and management remains limited to infection control and hemodynamic support.

N6-methyladenosine (m6A) is the most abundant internal chemical modification on mammalian mRNA. It is installed by a methyltransferase complex (writers: METTL3, METTL14, and cofactors such as WTAP, RBM15, and ZC3H13), removed by demethylases (erasers: FTO and ALKBH5), and functionally interpreted by reader proteins (YTHDF1/2/3, YTHDC1/2, IGF2BP1/2/3) that determine the fate of modified transcripts—stability, translation efficiency, splicing, or subcellular localization (7–9). The m6A modification is enriched around stop codons and within 3′-UTRs in a DRACH consensus motif, and its dynamic reversibility enables rapid transcriptomic reprogramming in response to stress (10).

In 2021, Wang et al. published the first systematic review linking m6A to sepsis-induced cardiovascular dysfunction (11). That review made an important conceptual contribution by organizing both direct and indirect evidence. However, the authors explicitly acknowledged that direct cardiac m6A evidence was limited at the time of writing, and much of the discussion relied on extrapolation from m6A roles in cardiac development, cardiomyopathy, or immune cells. Since 2022, the landscape has changed substantially. A growing body of original studies has since directly examined m6A regulators in cardiomyocytes or myocardial tissue under septic conditions, covering a range of writers (METTL3, METTL5, RBM15), erasers (FTO, ALKBH5), and readers (YTHDF1, YTHDF2, YTHDC1, YTHDC2, IGF2BP1). Several recent broad reviews have also discussed m6A in the context of sepsis or SICM (12–15), but the rapidly growing direct cardiac literature would benefit from a more focused synthesis that distinguishes injury-associated pathways from adaptive stress responses and that weighs the relative strength of current evidence.

This review organizes direct cardiac findings across four injury-associated processes—inflammatory injury, apoptosis, pyroptosis, and ferroptosis—together with the adaptive mitochondrial quality-control process of mitophagy (Table 1). We then integrate these data with emerging omics evidence and therapeutic implications, and identify the major translational gaps that must be addressed before m6A-targeted strategies can advance toward clinical application.

**Table 1:** Direct cardiac mechanistic studies of m6A regulation across key cellular processes in sepsis-induced cardiomyopathy.

Cellular process	Key finding	m6A regulator(s)	Principal target/pathway	Model	Study
Inflammatory injury	LPS suppressed cardiac FTO, increased global m6A, and raised cytokine mRNA methylation, aggravating myocardial inflammation and LV dysfunction	FTO (eraser)	Inflammatory cytokine transcripts; global m6A	LPS mouse; H9c2	(26)
Inflammatory injury	METTL3 catalyzed m6A on HDAC4 mRNA, and IGF2BP1 enhanced its stability, increasing HDAC4 expression and inflammatory injury	METTL3 (writer); IGF2BP1 (reader)	HDAC4	LPS H9c2	(27)
Inflammatory injury	YTHDC1 upregulation promoted myocardial inflammation and contractile dysfunction; silencing YTHDC1 reversed these effects	YTHDC1 (reader)	SERPINA3N	LPS mouse hearts	(28)
Apoptosis	METTL3-driven m6A promoted miR-193a maturation, suppressed anti-apoptotic BCL2L2, and aggravated cardiomyocyte apoptosis	METTL3 (writer)	miR-193a/BCL2L2	CLP mouse; LPS HL-1	(29)
Apoptosis	METTL3 knockdown improved cardiac function and reduced apoptosis and ROS through m6A-dependent regulation of Myh3 mRNA	METTL3 (writer)	Myh3	LPS rat; H9c2	(30)
Apoptosis	METTL3/YTHDF1 stabilized USP12 mRNA; USP12 deubiquitinated FOXO3, activating PUMA-dependent intrinsic apoptosis	METTL3 (writer); YTHDF1 (reader)	USP12/FOXO3/PUMA/caspases	LPS mouse; *in vitro*	(31)
Apoptosis	YTHDC2 promoted apoptosis and NF-κB activation, whereas cardiac-specific knockdown significantly attenuated injury	YTHDC2 (reader)	BAX/BAK1/NF-κB	SICM mouse; AAV9 cardiac-specific KD	(32)
Pyroptosis	ALKBH5 stabilized PTBP1 mRNA and promoted pyroptotic injury; knockdown of either ALKBH5 or PTBP1 attenuated myocardial dysfunction	ALKBH5 (eraser)	PTBP1	LPS rat; H9c2	(33)
Pyroptosis	RBM15-mediated m6A enabled YTHDF2-dependent SOX18 decay, increasing PTX3 accumulation and GSDMD-mediated pyroptosis	RBM15 (writer-complex component); YTHDF2 (reader)	SOX18/PTX3/GSDMD	SICM mouse; primary cardiomyocytes	(34)
Ferroptosis	METTL3 increased m6A on SLC7A11 mRNA, and YTHDF2 promoted its decay, impairing antioxidant defense and accelerating ferroptosis	METTL3 (writer); YTHDF2 (reader)	SLC7A11/GPX4	LPS H9c2	(35)
Ferroptosis	Silencing METTL3 suppressed ferroptosis through SLC7A11-related m6A regulation, supporting the METTL3/SLC7A11 axis	METTL3 (writer)	SLC7A11	Septic models	(36)
Ferroptosis	FTO overexpression suppressed ferroptosis via m6A-dependent regulation of BACH1; BACH1 overexpression reversed FTO-mediated protection	FTO (eraser)	BACH1	Septic animal and cell models	(37)
Ferroptosis	Atorvastatin downregulated METTL3 and inhibited the METTL3/IGF2BP1/CXCL2 axis, reducing inflammation and ferroptosis	METTL3 (writer); IGF2BP1 (reader)	CXCL2	SICM model; LPS HL-1	(38)
Ferroptosis	METTL5 promoted m6A-dependent YTHDF2-mediated NRF2 mRNA degradation, weakening antioxidant defense and accelerating ferroptosis	METTL5 (non-canonical writer); YTHDF2 (reader)	NRF2	Sepsis rat; LPS cardiomyocytes	(39)
Mitophagy	FTO promoted BNIP3-dependent mitophagy and preserved cardiac function by counteracting YTHDF2-mediated BNIP3 destabilization	FTO (eraser); YTHDF2 (reader)	BNIP3	LPS mouse; AAV9 cardiac-specific manipulation	(42)
Omics/landscape	Cardiac m6A remodeling was widespread in sepsis, with enrichment of IL-17- and MAPK-related pathways; findings remain hypothesis-generating	Global (WTAP, IGF2BP2)	859 differentially methylated genes	LPS mouse (MeRIP-seq)	(48)
Ferroptosis	ZC3H13 upregulation associated with ferroptosis in SICM; functional validation limited	ZC3H13 (writer complex)	Pnn/Rbm25/GPX4/SLC7A11	HL-1; dataset	(40)

CLP, cecal ligation and puncture; KD, knockdown; LPS, lipopolysaccharide; LV, left ventricular; SICM, sepsis-induced cardiomyopathy.

## 2 Overview of m6A RNA modification machinery

**Figure 1:**
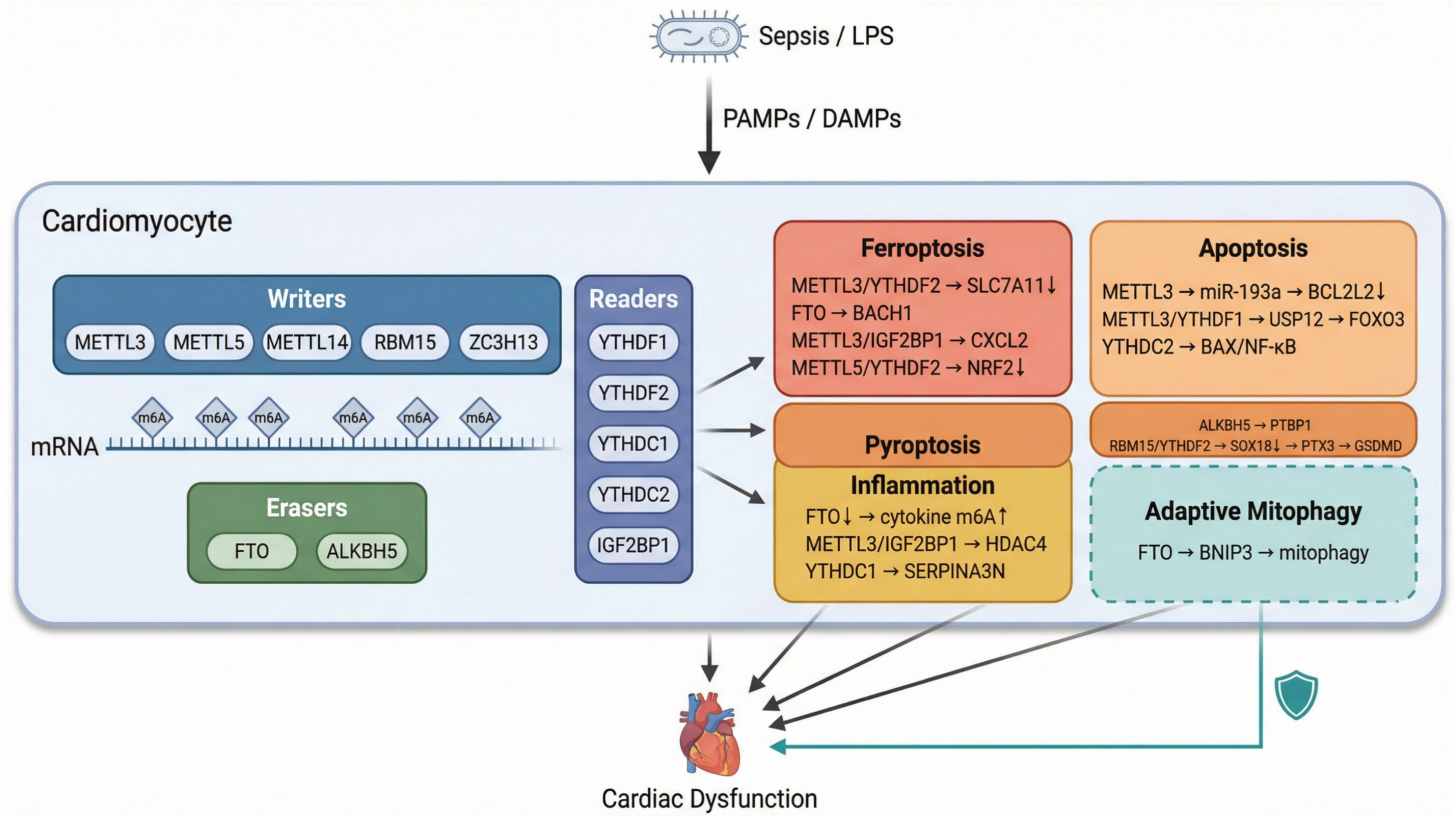
Direct cardiac m6A-regulated pathways across key cellular processes in sepsis-induced cardiomyopathy. The schematic summarizes experimentally supported writer-, eraser-, and reader-centered axes reported in cardiomyocytes or myocardial tissue under septic conditions. Four modules represent injury-associated processes (inflammatory injury, apoptosis, pyroptosis, and ferroptosis); the mitophagy module denotes an adaptive mitochondrial quality-control response.

The catalytic core of the m6A writer complex is formed by METTL3, which provides the S-adenosylmethionine-binding catalytic domain, and METTL14, which contributes to RNA substrate recognition and structural stabilization (16). Accessory proteins WTAP, RBM15/RBM15B, VIRMA, and ZC3H13 guide the complex to specific mRNA subsets and subnuclear compartments (17, 18). METTL5, a phylogenetically distinct methyltransferase, primarily catalyzes m6A on 18S rRNA, although recent evidence suggests additional mRNA targets (19). Two Fe(II)/α-ketoglutarate-dependent dioxygenases—FTO and ALKBH5—serve as erasers, conferring dynamic reversibility to m6A marks (20, 21). On the reader side, the YTH-domain family proteins perform distinct functions: YTHDF2 accelerates mRNA degradation, YTHDF1 promotes cap-dependent translation, YTHDF3 assists both processes, YTHDC1 regulates nuclear RNA splicing and export, and YTHDC2 facilitates translation or decay depending on context (22–24). The IGF2BP family (IGF2BP1/2/3) recognizes m6A-modified transcripts and stabilizes them against degradation (25). Thus, the functional consequence of m6A installation is not fixed but is determined by which reader engages the modified transcript and in what cellular context. Thus, the biological consequence of m6A installation is determined not simply by the writer or eraser involved, but by the combination of target transcript, reader engagement, and cellular context. This framework is essential for interpreting why some m6A pathways amplify myocardial injury whereas others support adaptive responses in SICM (Figure 1).

## 3 m6A in sepsis-induced myocardial inflammatory injury

Initial direct evidence for m6A involvement in septic cardiac inflammation showed that lipopolysaccharide (LPS)-induced endotoxemia increases global myocardial m6A levels while suppressing FTO expression, thereby enhancing methylation of inflammatory cytokine transcripts (IL-6, TNF-α, IL-1β), elevating cytokine protein levels, and impairing left ventricular function (26). These findings support the concept that the myocardial FTO–m6A balance shifts during sepsis toward a pro-inflammatory state.

Subsequent studies identified transcript-selective pathways superimposed on this global shift. METTL3-mediated m6A deposition on HDAC4 mRNA, together with IGF2BP1-dependent transcript stabilization, aggravated inflammatory injury in LPS-treated cardiomyoblasts (27). In parallel, upregulation of the nuclear reader YTHDC1 promoted SERPINA3N expression and worsened myocardial inflammation and contractile dysfunction in septic hearts; silencing YTHDC1 reversed these effects (28). This study extended the SICM-related m6A regulatory landscape to nuclear readers.

Collectively, these studies show that m6A-dependent inflammatory regulation in SICM operates at multiple levels: globally through the FTO–m6A balance, and at specific transcripts through parallel METTL3/IGF2BP1 and YTHDC1 axes. The protective role of FTO in restraining inflammation stands in contrast to the pro-inflammatory actions of writers and readers—a pattern of context-dependent regulation that recurs across other cellular processes examined below.

Taken together, current evidence suggests that m6A amplifies septic myocardial inflammation through at least two regulatory layers: a transcriptome-wide shift in the FTO–m6A balance and transcript-selective control by writer/reader pairs. An important conceptual implication is that m6A appears to function less as an initiator of pathogen sensing than as a post-transcriptional gain control on inflammatory output. However, because these data derive predominantly from LPS models and immortalized cell lines, whether the same regulatory hierarchy operates in polymicrobial sepsis or human myocardium remains uncertain.

## 4 m6A in sepsis-induced cardiomyocyte apoptosis

Apoptosis is the most extensively studied cell death program in the m6A–SICM literature, with multiple independent regulatory axes reported. Liang et al. (29) used a cecal ligation and puncture (CLP) mouse model combined with LPS-treated HL-1 cardiomyocytes to show that METTL3 promoted m6A-dependent maturation of miR-193a, which in turn suppressed the anti-apoptotic gene BCL2L2, driving cardiomyocyte apoptosis and inflammatory injury. In a parallel LPS–rat model, Gong et al. (30) found that METTL3 knockdown improved cardiac function and reduced apoptosis, ROS accumulation, and tissue injury, with evidence implicating m6A-dependent regulation of Myh3 mRNA stability in this phenotype.

Subsequent studies further delineated the mechanistic architecture of m6A-regulated apoptosis in SICM. A multi-step cascade was subsequently uncovered in which METTL3 and YTHDF1 cooperatively stabilized USP12 mRNA via m6A modification; USP12, a deubiquitinase, then prevented FOXO3 degradation, enabling FOXO3 to transcriptionally activate PUMA and trigger the intrinsic apoptotic pathway through BAX, Apaf-1, and caspases (31). Notably, pharmacological inhibition of the METTL3/YTHDF1/PUMA axis attenuated SICM *in vivo*, providing direct evidence for therapeutic targetability. In a complementary study, the reader YTHDC2 was shown to be was upregulated in SICM hearts and promoted both apoptosis (via BAX/BAK1) and NF-κB activation; AAV9-mediated cardiac-specific YTHDC2 knockdown significantly attenuated injury, and RIP-seq/RNA-seq analysis identified downstream target networks (32). The inclusion of cardiac-specific *in vivo* gene silencing, combined with multi-omics target discovery, provides relatively robust evidence for a cardiomyocyte-autonomous role of YTHDC2 in SICM.

These four axes converge on the apoptotic execution machinery but differ in their upstream m6A components: METTL3 serves as the initiating writer in three of four cases, yet cooperates with different readers (IGF2BP1, YTHDF1) and targets distinct mRNAs. This recurrence does not indicate that METTL3 acts through a single conserved apoptotic program; rather, it behaves as a stress-responsive upstream hub whose biological effect is specified by the particular transcript–reader axis engaged. Among the currently described processes, apoptosis is supported by comparatively stronger mechanistic depth, including cardiac-specific *in vivo* knockdown and multi-omics target discovery (32). This distinction is important when considering METTL3 as a therapeutic target, because broad catalytic inhibition may not uniformly reproduce the benefits observed for individual downstream axes.

## 5 m6A in sepsis-induced cardiomyocyte pyroptosis

Direct evidence linking m6A to cardiomyocyte pyroptosis remains limited to two studies, but the available data are conceptually informative. In one study, the eraser ALKBH5 was shown to increase PTBP1 mRNA stability in an m6A-dependent manner in LPS-treated rat hearts and H9c2 cells, thereby promoting pyroptotic injury; knockdown of either ALKBH5 or PTBP1 attenuated myocardial dysfunction (33). This finding is noteworthy because ALKBH5, as a demethylase, was expected to mirror the protective effects of the other eraser FTO. Instead, ALKBH5 exacerbated injury—a indicating that the two m6A erasers are not functionally interchangeable, and that the net effect of m6A removal depends on which specific transcripts are demethylated.

A second pyroptosis axis was identified using both *in vivo* SICM mouse models and primary cardiomyocytes (34). RBM15, a writer-complex component, mediated m6A installation on SOX18 mRNA. The reader YTHDF2 then recognized the modified transcript and promoted its degradation. Because SOX18 normally represses the pentraxin PTX3, SOX18 depletion led to PTX3 accumulation and subsequent GSDMD-mediated pyroptosis. The use of primary cardiomyocytes rather than immortalized cell lines enhances the physiological relevance of this finding.

The juxtaposition of these two studies underscores a principle that applies broadly across the m6A–SICM literature: the functional consequence of m6A modification is determined by the identity of the target transcript and the engaging reader, not by the writer/eraser classification of the upstream regulator.

Although the pyroptosis literature is still small, it makes two contributions to the broader m6A–SICM framework. First, the injurious effect of ALKBH5 establishes that demethylases cannot be assumed to be uniformly protective—a finding that contrasts sharply with the consistent cardioprotection observed for FTO. Second, the RBM15/YTHDF2/SOX18/PTX3 axis illustrates how writer–reader coordination can drive lytic inflammatory cell death through transcript-selective regulation. Together, these studies reinforce that functional direction in SICM is determined by target selection rather than by the nominal class of the upstream m6A regulator.

## 6 m6A in sepsis-induced cardiomyocyte ferroptosis

Ferroptosis is the most densely studied m6A-regulated death program in SICM, with several independent axes converging on the antioxidant defense system. The foundational study by Shen et al. (35) demonstrated that METTL3 catalyzed m6A modification on SLC7A11 mRNA—the gene encoding the cystine/glutamate antiporter xCT—and YTHDF2 subsequently promoted its decay. Loss of SLC7A11 compromised glutathione synthesis and GPX4-dependent lipid peroxide clearance, driving ferroptosis in LPS-treated H9c2 cells. Tang et al. (36) independently validated this axis, confirming that METTL3 silencing suppressed ferroptosis through SLC7A11 m6A methylation.

With respect to demethylase-mediated regulation, FTO overexpression was shown to suppress ferroptosis and improve myocardial injury and survival in septic models, acting through m6A-dependent regulation of BACH1 (37). This positions FTO as a consistent cardioprotective factor across inflammation (26), mitophagy (discussed below), and ferroptosis, reinforcing its candidacy as a therapeutic target.

Subsequent studies further expanded this ferroptosis-centered network. In addition, atorvastatin was reported to suppress the METTL3/IGF2BP1/CXCL2 axis, thereby linking pharmacologic intervention to m6A-regulated ferroptosis and inflammation (38). The broader therapeutic implications of this finding are discussed in Section 8.3. Because atorvastatin is already widely prescribed, these findings suggest potential translational relevance that warrants further investigation. Wang (39) identified a role for the non-canonical writer METTL5, which promoted m6A modification on NRF2 mRNA; YTHDF2 then mediated NRF2 mRNA degradation, collapsing the master antioxidant defense and accelerating ferroptosis. This study extends the m6A regulatory repertoire in SICM beyond the canonical METTL3/METTL14 complex (40). Across these studies, a convergent pattern emerges: multiple m6A axes independently impair the GPX4/SLC7A11/NRF2 antioxidant system, suggesting that ferroptosis-centered m6A regulation may represent the most coherent translational axis identified in SICM thus far.

Among all currently described processes, ferroptosis provides the strongest pattern of mechanistic convergence: independent studies implicate distinct m6A regulators, yet they repeatedly converge on the SLC7A11/GPX4/NRF2 antioxidant module. This reproducibility across separate axes increases confidence that the finding is not a study-specific artifact. At the same time, most data still derive from LPS-driven oxidative injury models, so the relative contribution of ferroptosis in clinically heterogeneous sepsis remains to be defined.

## 7 m6A in adaptive mitophagy and mitochondrial quality control

Unlike apoptosis, pyroptosis, and ferroptosis, mitophagy is not an injury program per se but an adaptive mitochondrial quality-control process that can preserve cardiomyocyte homeostasis under stress (41). It is included here because current SICM studies indicate that m6A regulation extends beyond injury execution to the modulation of compensatory mitochondrial responses. To date, only one study has directly addressed the role of m6A in mitophagy during SICM. Qi et al. (42) used LPS-challenged mice and AAV9-mediated cardiac-specific FTO manipulation to demonstrate that FTO promoted BNIP3 expression and mitophagy, thereby clearing damaged mitochondria and preserving cardiac function. When cardiac FTO was knocked down, dysfunction and apoptosis worsened; mechanistically, reduced FTO activity allowed YTHDF2 to destabilize BNIP3 mRNA through increased m6A modification. The use of cardiac-specific AAV9 knockdown provides relatively robust *in vivo* evidence for a cardiomyocyte-autonomous protective role of FTO.

When considered alongside its anti-inflammatory (26) and anti-ferroptotic (37) roles, FTO emerges as the most consistently protective m6A regulator in SICM, operating through at least three distinct downstream mechanisms. This contrasts sharply with ALKBH5, the other eraser, which promotes pyroptosis through PTBP1 (33). However, this consistency should not be generalized to all demethylases: ALKBH5 promotes pyroptosis through PTBP1 stabilization (33), underscoring that the biological direction of m6A removal remains transcript- and context-dependent.

## 8 Integrative perspectives on m6A regulation in SICM

### 8.1 Mechanistic convergence across key cellular processes

Three integrative themes emerge from the direct cardiac literature. First, m6A regulators should not be classified as intrinsically beneficial or harmful; their biological effects are determined by the combination of target transcript, reader engagement, and cellular context. Within the direct cardiac evidence reviewed here, FTO exhibits the most consistent protective profile—spanning inflammation (26), ferroptosis (37), and mitophagy (42)—whereas METTL3 is predominantly injury-promoting. Although broader sepsis evidence suggests that METTL3 may be protective in certain non-cardiomyocyte settings (43), extrapolation to the heart should be made cautiously.

Second, the strongest cross-study convergence occurs in ferroptosis, where distinct writer-, reader-, and eraser-centered pathways repeatedly impinge on the SLC7A11/GPX4/NRF2 antioxidant axis. This mechanistic coherence across independently conducted studies increases confidence that ferroptosis-centered m6A regulation represents a genuine pathological node rather than a study-specific artifact.

Third, the evidentiary weight across the field is uneven. Findings supported by cardiac-specific AAV9 manipulation (32, 42) or validated in primary cardiomyocytes (34, 44) should currently be accorded greater interpretive weight than those based solely on H9c2 or HL-1 cell lines.

Beyond direct cardiomyocyte mechanisms, limited evidence suggests that m6A may also influence SICM through immune–cardiac crosstalk (e.g., macrophage-derived exosomal signals (43, 45)) or vascular–cardiac interactions (46). Because these data do not constitute direct cardiac mechanistic evidence, they are best viewed as future directions rather than core support for translational prioritization.

### 8.2 Omics-based landscape of cardiac m6A remodeling

Global profiling studies suggest that septic myocardium undergoes broad epitranscriptomic remodeling. Epitranscriptomic microarray analysis first indicated widespread m6A changes in endotoxemic rat hearts (47), and subsequent MeRIP-seq/RNA-seq analysis identified 859 differentially methylated genes enriched in IL-17- and MAPK-related pathways in septic mouse myocardium (48). These datasets are valuable because they expand the candidate network well beyond the small number of mechanism-validated axes discussed above. However, they remain primarily hypothesis-generating; in most cases, altered methylation has not yet been linked to causal changes in transcript fate, protein output, or cardiac phenotype. Bioinformatic evidence implicating the writer-complex component ZC3H13 in SICM-associated ferroptosis should be interpreted within this preliminary tier until functional validation is strengthened (40). In our view, the most productive next step will be to prioritize omics candidates that overlap with already validated injury circuits, rather than to expand target lists without functional stratification.

### 8.3 Therapeutic implications and repurposing opportunities

Therapeutic translation currently rests on two tiers of evidence. The first involves clinically available agents that appear to modulate m6A-related injury circuits indirectly. Atorvastatin attenuated the METTL3/IGF2BP1/CXCL2 axis and reduced inflammation and ferroptosis in SICM models (38), whereas dexmedetomidine was reported to modify the METTL3/FTO–PRMT5 pathway and suppress ferroptotic injury (44). Because both drugs are already in clinical use, they may offer a pragmatic repurposing opportunity that can be evaluated more rapidly than *de novo* agents.

The second tier, still entirely theoretical in SICM, is direct pharmacologic targeting of m6A machinery. Proof-of-principle has been established in other disease settings with compounds such as the METTL3 catalytic-site inhibitor STM2457 (49), and a growing catalog of small-molecule modulators of m6A regulators is emerging (50). Whether such agents can be delivered safely and selectively during sepsis, however, remains unknown. Given the pronounced context dependence documented in this review, a pragmatic near-term strategy may be targeted interception of ferroptosis-centered m6A nodes rather than broad systemic manipulation of m6A signaling.

### 8.4 Current limitations and translational priorities

Several limitations constrain interpretation of the current evidence base. The majority of studies rely on single-dose LPS models, whereas polymicrobial CLP models remain underused. *In vitro* work still depends heavily on H9c2 or HL-1 cell lines, with limited validation in primary cardiomyocytes. Cardiac-specific genetic manipulation has been reported in only two studies (32, 42), leaving unresolved the extent to which observed phenotypes are cardiomyocyte-autonomous rather than secondary to systemic inflammation. No study has validated key m6A axes in human myocardial samples. Finally, the temporal dynamics of m6A regulation across the biphasic course of sepsis—early hyperinflammation versus late immunosuppression—remain entirely unexplored. Addressing these gaps will be essential before m6A-directed interventions can be considered clinically actionable.

## 9 Conclusion

m6A RNA methylation has emerged as an important post-transcriptional regulatory layer in SICM, influencing not only injury-associated pathways such as inflammation, apoptosis, pyroptosis, and ferroptosis but also adaptive mitochondrial quality control through mitophagy. Current direct cardiac evidence most strongly supports a protective role for FTO and identifies ferroptosis-centered m6A regulation as the most convergent translational axis. Future progress will depend less on cataloging additional targets and more on resolving cell-type specificity, temporal dynamics, and—ultimately—human relevance.
